# Rodent Models of Diabetic Neuropathy, Role of Calcium Homeostasis in Pain and KB-R7943 as a Potential Therapeutic

**DOI:** 10.3390/ijms26052094

**Published:** 2025-02-27

**Authors:** Natasha Ivanova, Milen Hristov, Pavlina Gateva

**Affiliations:** 1 Institute of Neurobiology, Bulgarian Academy of Sciences, 1113 Sofia, Bulgaria; 2 Department of Pharmacology and Toxicology, Faculty of Medicine, Medical University of Sofia, 1431 Sofia, Bulgaria; mhristov@medfac.mu-sofia.bg (M.H.);

**Keywords:** diabetic neuralgia, animal models, Na^+^/Ca^2+^ exchanger blocker, streptozotocin, alloxan, high-fat diet

## Abstract

Diabetic neuropathy (DN) is characterized by nerve damage as a consequence of diabetes mellitus. Diabetes causes high blood glucose and triglyceride levels, which destroy the nerve blood vessels over time and trigger DN. Peripheral neuropathy is the most common type of DN, which encompasses a broad range of symptoms. One fourth of patients with diabetes suffer from neuropathic pain, which decreases their quality of life and puts them at high risk for emotional disturbances and depression. Finding an adequate therapy is an essential element in the cure of painful DN (PDN). Since the pathophysiology of this disease still needs to be elucidated, this has led to the development of various in vivo diabetic models. Animal models of DN not only provide insights into this disease but also are significant drivers for treatment assessment and improvement. In this review, we present the major features of the most commonly used chemically and diet-induced models of PDN in rodents and their progress to date, which are utilized for a better understanding of the disease mechanism for finding novel therapeutics. Considering the role of Ca^2+^ homeostasis in pain, we also review our recent research data on the Na^+^/Ca^2+^ exchanger blocker KB-R7943, which is a potential neuropathic pain reliever in a rodent model of DN.

## 1. Introduction

Diabetic neuropathy (DN) is nerve damage caused by long-lasting high glucose and lipid levels in the blood as a consequence of diabetes mellitus [1]. It is the most common complication that occurs in patients with type 1 and 2 diabetes [1]. The global diabetes prevalence surpassed 800 million adults in 2022 worldwide [2]. Over half of diabetics gradually and slowly develop symptoms of DN, which affects the central and peripheral nervous systems [1]. This disease initially impacts the sensory neurons, leading to various types of symptoms, such as pain, aberrant sensation, nerve degeneration, and sensory decline over time with late-stage involvement of the autonomic and motor nerves causing dysfunction of the internal organs or motor deficiency [1,3,4]. Still, peripheral neuropathy prevails in people with diabetes, making up about 75% of diabetic neuropathies, and it represents a significant health and financial burden [5,6]. It predominantly manifests with sensorimotor deficits in the lower extremities, progressing slowly to ulcerations with an increased risk of amputation [1,3,7]. This deteriorates patients’ daily activities, and in severe cases, the disease can spread to the upper limbs, the trunk, and the whole body, which also impedes patients’ quality of life [1,3,7]. Nearly a quarter of diabetic patients with peripheral neuropathy develop painful symptoms [8].

Neuropathic pain is the cruelest symptom of peripheral DN, having a greater impact on the quality of life of patients with pain than those without pain and causing sleep disorders and depression development [3,7,8,9]. Diabetic neuralgia is a spontaneous pain that includes burning, tingling, needles and pins, numbness, allodynia, hyperalgesia, and paresthesia [9]. The pathophysiology of diabetic neuropathy involves a complex interplay of metabolic, vascular, and inflammatory factors that lead to nerve damage and pain [9]. The mechanisms contributing to the development of pain in diabetic neuropathy include hyperglycemia, which leads to oxidative stress and disrupts calcium (Ca^2+^) homeostasis in neurons and glial cells, causing Ca^2+^ overload, endoplasmic reticulum stress, and impaired mitochondrial function [9,10]. This results in neuronal hyperexcitability, hypersensitivity, neuronal dysfunction, and apoptosis. High blood sugar significantly contributes to the release of pro-inflammatory cytokines in the spinal cord and peripheral nerves, which promote inflammation and contribute to pain [9,11]. Oxidative and nitrosative stress in DN causes dysfunction of the mitochondrial Ca^2+^ uniporter, preventing proper Ca^2+^ uptake and further exacerbating Ca^2+^ overload in neurons, contributing to neuronal injury [9]. Hyperglycemia affects Schwann cells and axons, leading to demyelination, impaired nerve conduction, and the development of neuropathic pain [9]. The abnormal activation of nociceptive pathways in response to stimuli can lead to persistent pain sensations in diabetic neuropathy [9].

Assuming that the complex pathogenesis of PDN is not fully understood, its exploration could lead to successful therapy [12]. In vivo animal models of DN are an important tool for studying the disease and its complications, including pain, as well as for discovering and assessing the efficacy of new medical therapies. Mice and rats are preferred due to their low cost, different strains, ease of treatment and accessibility, as well as their anatomical and physiological similarities to humans [13]. A variety of protocols and techniques and their convenience have led to the implementation of numerous induced in vivo DN models suitable for studying the disease in humans [14]. There are three major classes of DN animal models: chemically, diet- and genetically induced ones. Alternative models like pancreatectomy surgery and virus-induced rodent models have also been proposed [15,16]. The alternative and genetic models, being more expensive and not broadly available for regular use, with further limitations and uncertainties that need to be clarified, are not covered in this review [15,16,17,18,19]. The increasingly used models of DN are two types, chemically and diet-induced, with some protocols using a combination of them [20,21]. However, the perfect prototype to completely mimic this human disease and to be cost-effective still needs to be found, requiring the existing models to be constantly revised and improved.

At present, antidepressants and anticonvulsants, the drugs of choice recommended for diabetic neuropathic pain, treat symptoms like allodynia and hyperalgesia rather than the cause [9]. The inadequate effect with pain reduction between 30% and 50% in DN patients and the side effects associated with the primary treatment reinforce the need for discovering new medications [8,22]. Scientific papers indicate that Ca^2+^ dysregulation has a significant role in the development of neuralgia [12]. In diabetic states, chronic neuronal hypercalcemia is implicated in the mechanism of nerve pain, causing oxidative stress and abnormal Ca^2+^ signaling, which result in neuronal hyperexcitability, making neurons more sensitive to stimuli and contributing to the painful sensations [12]. The application of Ca^2+^ modulators has been used to diminish pain hypersensitivity [12]. In this respect, our recent study investigated KB-R7943, a Na^+^/Ca^2+^ exchanger (NCX) blocker that has been proposed as an alternative remedy for diabetic neuralgia [23]. The drug, which primarily inhibits the NCX reverse mode (NCXrev) and decreases intracellular Ca^2+^, exhibited positive effects in managing neuropathic pain and associated depressive symptoms in streptozotocin (STZ)-induced diabetes in rats [23].

The purpose of the review is first to emphasize the key characteristics of the most frequently used drug- and diet-induced in vivo rodent models, their progress to date, and their role in studying painful neuropathy caused by diabetes mellitus, which will contribute to the discovery and efficacy evaluation of new medications. Secondly, by briefly discussing Ca^2+^ dyshomeostasis in diabetic neuralgia, we introduce our recent exploration of the NCX blocker KB-R7943 as an unconventional cure for diabetic neuralgia, the commonest and severe condition of DN, investigated in a rat model of painful DN.

## 2. In Vivo Animal Models of DN: Drug- and Diet-Induced

Both type 1 and type 2 diabetes are characterized by hyperglycemia with distinct pathophysiologies. In type 1 diabetes, the higher glycemia results from autoimmune damage of the pancreatic beta-(β)-cells, whereas in type 2 diabetes, it is caused by insulin resistance followed by dysfunction of the β-cells [24]. The progression of both diabetes types leads to complications, including DN, which is a disabling and painful condition characterized by damage to the nervous system [4]. The major part of the data regarding the pathogenesis and treatment of DN is derived from rodent models of prediabetes, type 1 and type 2 diabetes. Although rat and mouse models cannot fully replicate the disease in humans, they exhibit key features of human pathology, such as allodynia, progressive motor and sensory loss and nerve structure pathology (Table 1). According to Neurodiab recommendations, the following criteria should be evaluated to define DN: response to noxious/painful stimuli, nerve conduction velocity and/or altered nerve structure [25]. DN in rats and mice begins with a metabolic phase characterized by nocifensive behavior and slowed nerve conduction velocity, both of which are relatively reversible by medical intervention. This progresses to the chronic structural phase, reflecting changes in the peripheral nervous system [25].

Nowadays, two main methods are actively used to induce DN: diet- and drug-induced, which are preferred for their ease of maintenance and cost-effectiveness (Table 1). These models help researchers explore pain mechanism in DN and test new drugs. Typically, high doses of chemicals are used to induce type 1 diabetes, while nutrition alone or combined with low doses of chemicals is used to induce type 2 diabetes. The characteristics, main advantages and disadvantages of each model are summarized in Table 1.

### 2.1. Chemically-Induced DN Rodent Models

Two main substances are used for the induction of DN: STZ and alloxan. Both are glucose analogues that cause destruction of the β-cells, leading to insulin deficiency and higher glycemia, which is a pathology that resembles human type 1 diabetes mellitus [36].

#### 2.1.1. Streptozotocin (STZ)-Induced Diabetes

Streptozotocin (STZ), N-(methylnitrosocarbamoyl)-α-d-glucosamine, a naturally occurring derived from the Gram-positive bacterium Streptomycetes achromogenes, is the most wildly used diabetogenic chemical [36]. It selectively enters pancreatic islets b-cells via the glucose transporter GLUT2, causing alkylation of the DNA, the generation of free radicals that brings about DNA damage and abnormal Ca^2+^ homeostasis, which leads to rapid cells death, hyperglycemia and cachexia [12,36,37]. It can be administered intraperitoneally or intravenously through single or multiple injections [24].

The high dose for rats ranges from 35 to 60 mg/kg/day, while for mice, it ranges from 100 to 200 mg/kg/day, depending on the strain [20]. Higher doses causes hyperinsulinemia and severe hypoglycemia within the first hours after injection, followed by subsequent insulinopenia and elevated glucose levels, both of which are associated with high mortality rate [38,39]. Death can be overcome with STZ injection into fed animals or providing food or insulin after the injection [38,39].

Multiple low doses, a model of insulinitis, at approximately 40–50 mg/kg/day for 5 consecutive days may be less toxic in mice, but despite sustained hyperglycemia, some strains do not display the neuropathy phenotype [15,38].

The STZ DN models (Table 1) are cost-efficient, easy to maintain and exhibit a comparative reproducibility of human DN pathology [4,38]. The early signs of diabetic neuropathy (hypersensitivity progressing to hyposensitivity and slow nerve conduction velocity) occur within the first month after the onset of diabetes, while late structural changes of the nerves can be expected at 8–12 weeks or, in some strains, months after STZ application [4,25,38].

A disadvantage of the STZ drug is that it can be toxic to other organs [24], although this toxicity is not responsible for neuropathy development [4]. Other limitations include high mortality and lack of severe neuropathy complication, such as demyelination (Table 1) [38,40].

In the STZ model, several pathophysiological processes contribute to neuropathy and pain. In hyperglycemia-induced oxidative stress, high glucose levels promote the formation of reactive oxygen species (ROS), which lead to oxidative damage in nerves, including dorsal root ganglia neurons [41]. In endoplasmic reticulum stress, the accumulation of unfolded proteins due to hyperglycemia triggers ER stress, which disrupts Ca^2+^ storage in the ER, leading to Ca^2+^ overload in neurons and glial cells. This disrupts normal cellular function and promotes neuronal apoptosis [10]. Mitochondrial dysfunction occurs in STZ-induced diabetic models, including Ca^2+^ overload in mitochondria due to the impaired mitochondrial Ca^2+^ uniporter, which leads to reduced adenosine triphosphate production and compromised neuroprotective functions, promoting neuronal death [41]. In neuroinflammation, microglial activation and pro-inflammatory cytokine release in the spinal cord and peripheral nerves lead to the activation of nociceptors, increasing pain sensitivity that contributes to pain and neurodegeneration [42]. Calcium dysregulation in neurons and glial cells (including the spinal cord and dorsal root ganglia) contributes to neuronal hyperexcitability, which is a key mechanism behind pain hypersensitivity [42].

#### 2.1.2. Alloxan-Induced Diabetes

Alloxan, 2,4,5,6-tetraoxypyrimidine; 5,6-dioxyuracil, is an organic compound widely used for diabetes induction [43]. As a cytotoxic glucose analogue, it enters β-cells either via GLUT2 or through diffusion [43]. It inhibits the enzyme glucokinase, a pancreatic glucose sensor, and additionally generates reactive oxygen species (ROS) and induces DNA fragmentation, leading to β-cells apoptosis and type 1 diabetes mellitus [24]. The drug is also associated with thiol groups oxidation, including those of glucokinase and other thiol-enzymes, as well as Ca^2+^ dysregulation—effects that further contribute to the islet β-cells destruction [24].

Alloxan can be applied intraperitoneally or intravenously at a dose range of 40–200 mg/kg for rats and 50–200 mg/kg for mice [24].

Transient hypoglycemia is observed within minutes after alloxan administration, followed by insulin deficiency and hyperglycemia 1 h after the drug injection, which contributes to increased animal mortality [43].

The model is cost-effective, easy to conduct, and primarily mimics the early changes of DN (Table 1) [17,43].

Despite its relatively low cost, a limitation of using alloxan as a diabetogenic agent is that it is less effective than STZ, showing auto-reversibility from established diabetic hyperglycemia to a non-diabetic state [43]. Another unfavorable event is the elevated mortality due to hypoglycemic shock upon injection [43]. Since glucose competes with alloxan for GLUT2 binding, thereby lowering its accumulation in the cells, it has shown a protective effect against the alloxan induction of diabetes [43,44,45]. However, unsuccessful attempts have been made to reduce mortality by administering 5% or 10% of glucose solution [43,44,45]. The drug also has a tighter diabetic dose window, and small dose biases can lead to general toxicity [36]. In comparison with STZ, alloxan is more unstable, ineffective in some species, and has a narrower diabetic dose range, a shorter induced-hyperglycemia duration (up to 1 month), and less severe neuropathy, making it less desirable than STZ (Table 1) [46].

The mechanisms of neuropathy and pain in alloxan-induced diabetic models are similar to those in STZ models with some distinct features. First, there is oxidative stress. Alloxan itself generates reactive oxygen species, which directly contribute to oxidative damage in neurons and Schwann cells [24,47]. There are also metabolic changes. Similar to STZ, elevated blood glucose levels impair vascular function, leading to poor blood flow to nerves. This ischemia further exacerbates neural injury and contributes to neuronal damage [14,17,28,48]. Last, there is endoplasmic reticulum stress and Ca^2+^ dysregulation. High glucose levels activate the unfolded protein response in the endoplasmic reticulum, causing Ca^2+^ leakage and disrupting the Ca^2+^ homeostasis within neurons and glial cells. This Ca^2+^ overload in the cytosol leads to neuronal dysfunction, hyperexcitability, and eventually cell death [49]. Oxidative stress and an excess of Ca^2+^ result in nociceptor sensitization and hyperexcitability, making neurons more responsive to stimuli.

### 2.2. Diet-Induced DN Rodent Models

Genetic factors, combined with a sedentary lifestyle and the consumption of unhealthy, high-calorie foods, contributing to weight gain, are main triggers for prediabetic state or type 2 diabetes [50]. An ongoing increase in the body mass index leads to metabolic changes, hyperinsulinemia and insulin resistance [19,51]. Obesity-induced impaired insulin sensitivity typically results in β-cell dysfunction and impaired glucose homeostasis, all of which are hallmarks of type 2 diabetes mellitus, affecting 90 percent of the human diabetic population [19,51]. In this context, the majority of the type 2 diabetes animal models are obese, attempting to more accurately reflect the disease in humans [24]. Neuropathy is a serious health problem in both people with prediabetes or type 2 diabetes [32,52]. The nutritional animal models that are most widely used in DN research include high-energy diets (high-fat-diet, high-sugar-diet and different combinations of them) (Table 1).

Using a high-fat-diet, in 2007, Obrosova et al. developed a neuropathy model in female C57BL6/J mice [32]. Diet-induced obesity replicates features of prediabetes and metabolic syndrome, both of which are associated with diabetic neuropathy development. In these models, rodents are fed food enriched with fat, sugars or a combination of them for induction of obesity following different protocols based on the type and percentages of fat/sugar content and the duration of feeding [15,17,30,35]. It is worth mentioning that the various procedures, along with factors such as strain, sex and age, can influence the time for neuropathy to develop and its severity [15,30,35,53]. Usually, saturated fats are used with 25–60% of the food content, while carbohydrates can vary between 10% and 80% of the total composition [53]. The C57/BL6 mouse strain is highly susceptible to diet-induced obesity and is widely used to model obesity-related diabetic neuropathy changes [30], similar to rats fed a high-fat diet [29,35]. Both mice and rats fed a high-fat diet, either alone or combined with carbohydrates, exhibit weight gain, high plasma fatty acids, hyperinsulinemia, and impaired glucose tolerance but without frank hyperglycemia [29,30,32,33]. Neuropathy-related nerve dysfunction typically becomes evident after at least eight weeks of feeding, manifesting as decreased motor and sensory nerve conduction velocity, allodynia, early hyperalgesia and later hypoalgesia. However, structural markers of neuropathy, such as decreased intraepidermal nerve fiber density, require a longer duration to develop (Table 1) [15,30].

A disadvantage of these models is the longer time required for the neuropathic changes to appear as well as the need for the regular monitoring of food intake. A key benefit of the diets is that environmental manipulations closely mimic disease development in humans. In general, these models are suitable to study insulin-resistant and prediabetes neuropathy.

### 2.3. Combined Chemically- and Diet-Induced DN Rodent Models

Some authors have advised that using a combination of diet and drug models to induce type 2 diabetes results in neurological impairments that more accurately reflect features of the human disease [20,35,54]. While high doses of STZ produce severe hyperglycemia and type 1 diabetes, low to moderate doses of the drug combined with a high-energy diet are used to induce type 2 diabetes and neuropathy in rats and mice [30]. Diet composition and duration, as well as the STZ doses, vary across investigations [15,34]. Typically, a high-energy diet course provokes insulin resistance, while low to moderate doses of STZ generate moderate hyperglycemia and only partially destroy pancreatic β-cells [15,34].

These models gather attention because they are less toxic and provoke the gradual development of type 2 diabetes and neuropathy changes in both mice and rats (Table 1).

The overconsumption of high-energy foods causes an increase in blood glucose levels, which triggers insulin resistance and hyperinsulinemia. Elevated glucose levels promote oxidative stress, advanced glycation end-product formation, and inflammation, all of which contribute to neuropathy [55]. Calcium overload in neurons and glial cells due to mitochondrial dysfunction, similar to the chemically-induced DN models, leads to neuronal apoptosis and loss of neuroprotective functions [10,56]. This contributes to the sensitization of pain pathways and increases the perception of pain in diabetic neuropathy models induced by high-energy diets [17]. In these models, pro-inflammatory cytokines are upregulated in the peripheral nerves and spinal cord, which promotes glial activation and worsens the inflammatory environment, contributing to pain hypersensitivity and the hyperexcitability of nociceptive pathways [57].

## 3. Calcium Dyshomeostasis in Diabetic Neuralgia

Calcium homeostasis is crucial for normal nerve function, including neurotransmitter release, signal transduction, and neuronal excitability [58]. Investigations have confirmed the critical role of Ca^2+^ homeostasis in the pathogenesis of DN and diabetic nerve pain [12,37,59]. Abnormal Ca^2+^ signaling in neurons and many other tissues have been identified in both animal models of diabetes and diabetic patients [12,60]. Increased Ca^2+^ concentration in diabetes, including DN and diabetic neuralgia, has been attributed to numerous factors: abnormalities in the expression and physiology of Ca^2+^ channels, the aberrant work of sodium–potassium pump (Na^+^/K^+^-ATPase) that modulates the activity of NCX [60], impaired endoplasmic reticulum rapid Ca^2+^ release and maintaining, increased activity of N-methyl-D-aspartate (NMDA) and alpha-amino-3-hydroxy-5-methylisoxazole-4-propionicacid (AMPA)/kainite receptors and altered extrusion systems work (plasmalemmal Ca^2+^-ATPase pump and Na^+^/Ca^2+^ exchanger (NCX)) [12,59].

In experimental models of DN, including in STZ- or alloxan-induced diabetes in rats, neuronal cytosolic Ca^2+^ overload produces nerve injury, which results in pain, mitochondrial dysfunction, axonal degeneration and cell death [12,43,60,61]. Chronic hyperglycemia induced by STZ and high-energy diets increases endoplasmic reticulum stress and disrupts Ca^2+^ storage in the endoplasmic reticulum [10]. This results in Ca^2+^ leakage into the cytoplasm, which contributes to intracellular Ca^2+^ overload [10]. This overload affects neuronal function, leading to hyperexcitability and pain hypersensitivity [10]. Streptozotocin induces the overactivation of T-type (Cav3.2) and L-type (Cav1.2) Ca^2+^ channels in nociceptive neurons, particularly in dorsal root ganglia neurons [62,63]. These channels are important for transmitting nociceptive signals. In diabetic models, these channels become hyperactive, allowing excessive Ca^2+^ influx, which leads to neuronal hyperexcitability [62,63]. This is characterized by an increase in the action potential firing rate and reduced pain threshold, both of which are crucial contributors to mechanical allodynia and thermal hyperalgesia in diabetic neuropathy. In STZ-induced diabetes, not only nociceptive neurons but also astrocytes experience Ca^2+^ overload due to both increased Ca^2+^ influx and decreased buffering capacity [64]. This leads to the release of pro-inflammatory cytokines (e.g., TNF-α, IL-6), which in turn activate nociceptive pathways, contributing to pain [64]. Diabetes models, including STZ, alloxan or high-energy diet models, show increased microglial activation and the release of pro-inflammatory mediators, which sensitize neurons and exacerbate pain [42]. The excess Ca^2+^ in glial cells, particularly microglia, leads to the activation of inflammatory pathways, which damages the glial cells [42].

Damage of the sensory nerves in DN is linked with uncommon pain sensation [12]. Although the exact mechanism of PDN is not fully understood, one of the main reasons is thought to be chronic hyperexcitability of the sensory neurons secondary to Ca^2+^ signaling changes, leading to elevated Ca^2+^ cell levels responsible for the disruption of a large variety of nerve functions that gives rise to sustained pain [12].

Diabetic neuropathic animal models provide strong evidence that Ca^2+^ dysregulation is a key driver of pain hypersensitivity and neuronal dysfunction in PDN. STZ and alloxan induce Ca^2+^ ion overload in both nociceptive neurons and glial cells, disrupting Ca^2+^ homeostasis and triggering neuronal hyperexcitability, neuroinflammation, and cellular dysfunction. This cascade of events leads to the onset and maintenance of PDN.

## 4. Na^+^/Ca^2+^ Exchanger (NCX) in PDN

**Figure 1:**
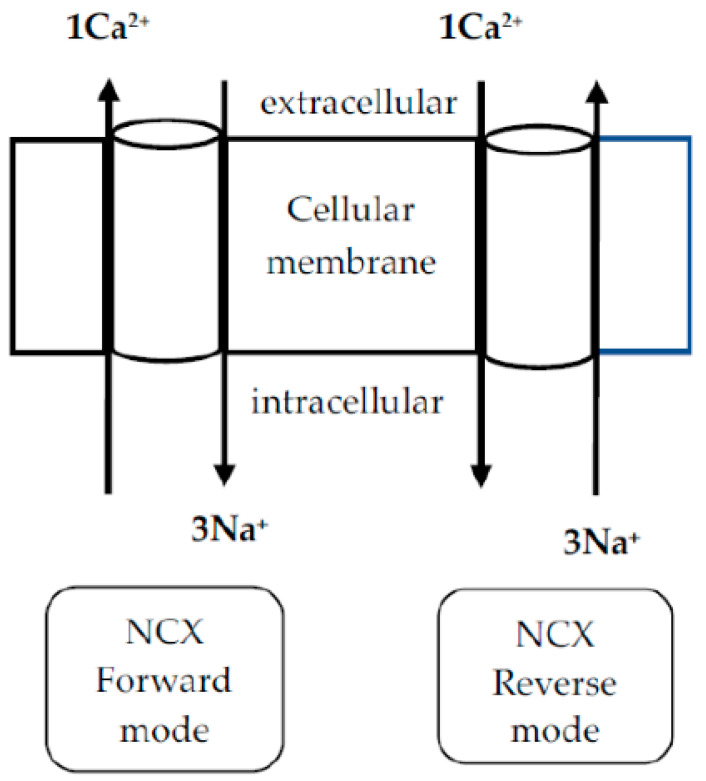
Schematic presentation of the function of the Na^+^/Ca^2+^ exchanger [66].

The NCX, a Ca^2+^ transporter that controls the intracellular Ca^2+^ concentration across the cell membrane, is another significant pathway for Ca^2+^ entry that worth attention in degenerative and PDN. This exchanger helps maintain intracellular Ca^2+^ levels and plays a vital role in processes such as excitation–contraction coupling, signal transduction, and cardiac and neuronal function [65]. This protein operates in a bidirectional way, exchanging 3 Na^+^ for 1 Ca^2+^ ion (Figure 1, [66]) and whilst operating in its reverse mode could become a reason for intracellular Ca^2+^ overload, which is linked to diverse pathological states [65].

It exists in different cells and has three isoforms, NCX1, NCX2 and NCX3, which are also expressed in central and peripheral neurons and glial cells [65]. The differences between the three isoforms are described in Table 2.

Only a few studies have raised the question about the role of the NCX in diabetic conditions [67,68,69,70,71]. It operates in reverse mode in hyperglycemic states, accelerating cellular Ca^2+^ overload, which ends in platelet hyperactivity and glucose-induced endothelial dysfunction [68,69]. A common complication of diabetes and DN is electrolyte imbalance, including hyponatremia [70,71], which by blocking the Na+/K+ pump can reverse the operation of NCX and release Ca^2+^ influx into cells [72].

All these data suggest that regulation of the Ca^2+^ homeostasis is of supreme importance in managing the DN complexity, including its painful symptoms. Numerous current and newly investigated drugs designated to alleviate PDN have restricted use due to the side effect profiles, route of administration, specific molecular target, addiction and other concerns [73,74,75,76]. Finding a curative therapy with reduced side effects that can prevent or achieve DN pain relief remains highly relevant. In this regard, inhibiting NCXrev is an attractive drug therapy approach that could prevent Ca^2+^ overload, excitotoxicity, and the resulting painful condition.

## 5. KB-R7943 in the Treatment of PDN

### 5.1. Other Therapeutic Targets and Drugs for PDN

Several pharmacological approaches are commonly used to treat PDN. Tricyclic antidepressants (amitriptyline) and serotonin–norepinephrine reuptake inhibitors (duloxetine) are often used for managing neuropathic pain. These drugs work by modulating central pain processing and neurotransmitter systems like serotonin and norepinephrine [77]. Gabapentin and pregabalin are anticonvulsants that act on voltage-gated Ca^2+^ channels. They reduce Ca^2+^ influx into neurons, which decreases neuronal excitability and can alleviate neuropathic pain. These drugs are commonly used for PDN and other neuropathic pain syndromes [77]. Sodium channel blockers like carbamazepine, oxcarbazepine, lacosamide and lamotrigine inhibit the activation of nociceptive pathways in the peripheral and central nervous systems, offering relief from neuropathic pain [78]. Opioids (morphine, oxycodone) are sometimes prescribed for severe pain, but their use is limited due to potential for dependency and side effects [5,8]. Topical agents like capsaicin or lidocaine patches provide localized relief by desensitizing nerve endings and reducing pain signaling [8]. Agents like alpha-lipoic acid and vitamin E have been studied for their potential to reduce oxidative stress and inflammation, which are important contributors to the development of diabetic neuropathy [79].

Given that current pharmacological therapies display various side effects and an adequate treatment goal has not been achieved yet, here is a pressing need to develop new and more effective treatments for DPN.

### 5.2. Current Challenges and Future Directions

**Figure 2:**
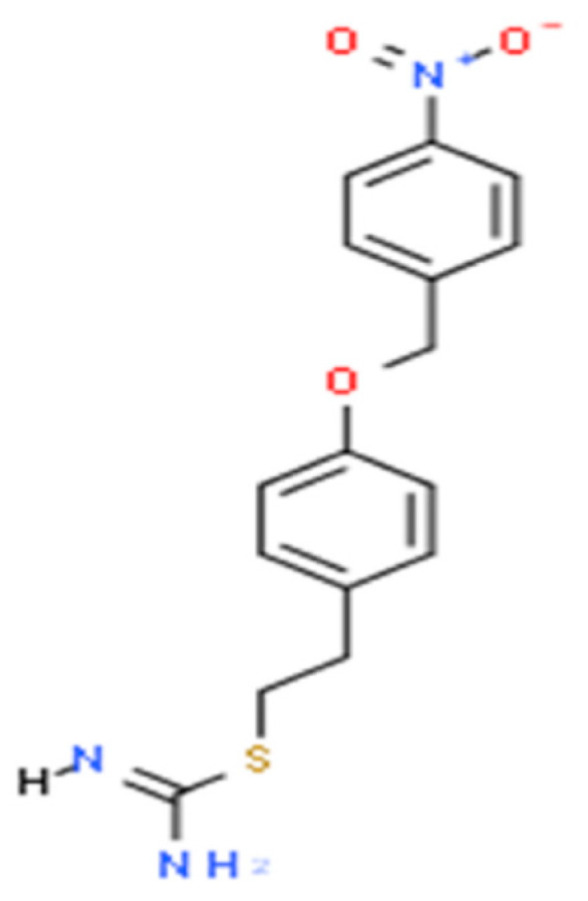
Chemical structure of KB-R7943 (data from ChemAxon).

KB-R7943 was discovered and developed as part of efforts to find NCX inhibitors that could modulate intracellular Ca^2+^ levels in cells [66]. The discovery was driven by the need to develop compounds that could block the Na^+^/Ca^2+^ exchanger, which plays a critical role in various diseases where Ca^2+^ overload is a significant factor. The compound was first synthesized by Shigekawa’s group [66]. The primary goal was to find a molecule that could specifically block NCX activity without affecting other Ca^2+^ channels or ion transporters in the body, which could lead to unwanted side effects. KB-R7943 is an isothiourea derivative 2-[2-[4-(4-nitrobenzyloxy)phenyl]ethyl]isothiourea methanesulfonate with a molecular weight of 372 and chemical structure shown in Figure 2 (data from ChemAxon).

It nonspecifically inhibits the reverse mode of the three NCX isoforms, with greater affinity to NCX3, by binding to a specific site on the NCX protein [63]. The isothiourea group is typically protonated, which is crucial for its inhibitory activity. The distinct inhibitory effects of KB-R7943 imply that the benzyloxy group plays a key role in determining selectivity between different isoforms [63]. As a prominent inhibitor of the NCX, the role of KB-R7943 in preventing cell Ca^2+^ overload has been demonstrated not also by targeting the NCXrev but also by inhibiting L-type voltage-gated Ca^2+^ channels, store-operated Ca^2+^ influx, mitochondrial Ca^2+^ uptake, transient receptor potential channels, NMDA receptors and mitochondrial respiratory chain complex I [63,80]. Moreover, the drug is cable of blocking voltage-sensitive Na^+^ currents, L-type Ca^2+^ currents, and inward K^+^ currents in cardiomyocytes [63]. It has been found to be effective in reducing brain injury triggered by ischemia–reperfusion [81], suppressing seizures [82], decreasing neuronal death promoted by hypoxia/hypoglicemia ischemic episodes [83] or mechanical brain trauma [84], and mitigating glutamate mediated excitotoxicity in the brain [85]. Although promising, NCX inhibition is a double-edged sword because an excessive inhibition of NCX could lead to Ca^2+^ overload in non-neuronal cells like cardiomyocytes [63]. This might cause arrhythmias or cardiac dysfunction. Hence, future research must balance the efficacy of NCX blockers with their safety profile [63].

Considering the contribution of NCX to Ca^2+^ dyshomeostasis and Ca^2+^ excess associated with the mechanism of neuropathic pain, and the important effects of KB-R7943, our recent research focused on testing the effects of KB-R7943 against diabetic neuralgia and related depression [23]. Diabetes was induced via a single injection of STZ in adult male Wistar rats. The animals that developed panful symptoms, validated by the cold plate, paw pressure and formalin tests conducted approximately one month post SZT injection, were used for the KB-R7943 investigation. Two doses of the drug were tested and applied for a one-week period: 5 and 10 mg/kg,/once daily, administered via gavage, chosen on the basis of reported decreased seizure susceptibility [86]. The 10 mg dose of KB-R7943 alleviated mechanical allodynia and anti-nociceptive behavior (licking and biting), as demonstrated in the paw pressure and formalin tests, effects similar to those of amitriptyline, which is used as a reference drug. To our knowledge, this is the first study to test KB-R7943 against neuropathic pain due to diabetes mellitus. Additionally, the drug has been found to partially counteract axonopathy of the sensory neurons induced by linezolid treatment [87]. Counting the role of NCX in pain, these data suggest that the positive outcomes of KB-R7943 are primarily attributed to its ability to inhibit the NCXrev. Therefore, along with its additional Ca^2+^-modulating activities, the drug prevents Ca^2+^ excess and subsequent cytotoxicity involved in the progress of neuropathic pain.

We also showed that the higher dose of KB-R7943 (10 mg/kg) was able to reverse the depression-like behavior in DN rats, which was tested in the forced swimming test [23]. Given that pain from neuropathy is often accompanied by emotional disturbances, including depression, we hypothesize that the alleviation of the painful condition by the drug, as mentioned above, could explain its beneficial impact on depression associated with diabetic neuropathic pain.

Our results suggest that KB-R7943, a potent inhibitor of NCXrev mode, may be beneficial in treating PDN and associated complications, warranting additional studies to further explore the potential benefits of the drug.

While KB-R7943 has shown promise in animal models, including our own, clinical translation is still in its early stages. The selectivity, potency, and long-term safety of this compound need further clinical validation before widespread therapeutic use.

## 6. Conclusions

To be appropriate, the DN model should represent as many aspects of DN pathology in humans as possible and be drug-validated, which remains a challenge. Streptozotocin, alloxan, and high-energy diet animal models of diabetic neuropathy share several key characteristics with human PDN. These models develop chronic hyperglycemia, just like in human diabetes, leading to oxidative stress, inflammation, and nerve damage. Animals with diabetic neuropathy show axonal degeneration, demyelination, and reduced nerve conduction velocity, which mirror the pathological changes seen in human patients. Common pain symptoms in human PDN, such as spontaneous pain, mechanical allodynia, and thermal hyperalgesia are also observed in animal models. Just like in humans, diabetic animals exhibit increased pro-inflammatory cytokines (e.g., TNF-α, IL-6) and the activation of immune cells, which contribute to neuropathic pain. Studies show that both human and animal models experience sensitization of peripheral nerves and spinal cord neurons, leading to an exaggerated pain response. Mitochondrial abnormalities and oxidative stress play a significant role in both human and animal models, contributing to nerve damage and pain hypersensitivity. These models are widely used to study diabetic neuropathy and pain mechanisms, as Ca^2+^ dysregulation contributes to neuronal dysfunction, hyperexcitability, and pain hypersensitivity.

The important characteristics of the DN models reviewed in this paper display variations regarding mimicking this state in humans and still need to undergo treatment proof. Despite these limitations, preclinical models are undoubtedly invaluable in understanding the pathological mechanisms of DN and neuralgia. Continued efforts to improve these models will advance our understanding of the disease, which will pave the way for better treatment management.

In this paper, we also discussed how abnormal neuronal Ca^2+^ concentration is implicated in the pathogenesis of DN and neuropathic pain. Diabetic models are associated with Ca^2+^ dyshomeostasis, which triggers neuronal hyperexcitability, neuroinflammation, and cellular dysfunction. This cascade of events leads to the onset and maintenance of diabetic neuropathic pain. Understanding the role of Ca^2+^ dysregulation in diabetic neuropathy not only helps explain the pathophysiology of pain but also provides potential targets for therapeutic intervention.

Despite the availability of various therapies, current options often provide limited relief and are associated with significant side effects. Given the complex and multifactorial nature of PDN, treatments that target the underlying mechanisms of nerve damage and dysfunction—such as dysregulated Ca^2+^ homeostasis—are crucial. The important role of the NCX in regulating Ca^2+^ homeostasis has been highlighted. Operating manly in its reverse mode in diabetic states, the exchanger facilitates excessive Ca^2+^ influx, leading to excitotoxicity in the nerve cells. We proposed an alternative treatment of diabetic neuropathic pain by targeting the reverse mode of NCX. KB-R7943, as an NCXrev inhibitor, can prevent Ca^2+^ overload and nerve toxicity progressing to diabetic neuralgia. The drug has demonstrated promising effects as a diabetic nerve pain reliever. Based on our results, KB-R7943 might represent a surrogate or an adjuvant option for managing complications in DN. The identification and development of novel therapies, particularly those targeting specific molecular pathways like the Na^+^/Ca^2+^ exchanger, could offer more targeted, safer, and more effective solutions for managing PDN and improving the quality of life for patients. With the increasing prevalence of diabetes, advancing our understanding of these mechanisms and discovering innovative treatments is more important than ever.

## Figures and Tables

**Table 1:** Chemically- and diet-induced rodent models of DN: features, advantages and disadvantages.

Method	Model	Features of DN			Advantages	Disadvantages
**Chemically-induced** (type 1 diabetes)	**STZ (single high doses or multiple low dose)**	**Nerve conduction velocity**	**Behavior**	**Nerve structure**		
	Rats (single high doses of 35–60 mg/kg/day) [14,17,26,27]	Decreased sensory and motor conduction velocity	Allodynia; hyperalgesia to hypoalgesia	Axonal degeneration; decreased intraepidermal nerve fiber density; reduced corneal nerve fiber length; Schwann cell proliferation; reduced right and left fascicles of phrenic nerve; molecular changes of the peripheral nerves	Low cost; fast induction of diabetes; known duration of diabetes and DN development	High mortality rate; neuropathy phenotype variability; cell/organ toxicity; lack of severe neuropathy changes resembling human pathology; still to elucidate DN pathogenic aspects
	Mice (single high doses of 100–200 mg/kg/day or multiple low doses of 40–50 mg/kg/day) [15]	Slow sensory and motor conduction velocity	Allodynia; hypoalgesia	Decreased intraepidermal nerve fiber density; decreased size of dorsal root ganglia neuron; molecular changes of the peripheral nerves	Low cost; fast induction of diabetes; known duration of diabetes and DN development	Toxicity and late development of neuropathy in the single high-doses model; lack of severe neuropathy changes resembling human pathology; lack of neuropathy feature of some strains in the multiple low dose
	**Alloxan**					
	Rats and mice (single doses of 40–200 mg/kg for rats and 50–200 mg/kg for mice) [14,17,28]	Slow nerve conduction velocity	Allodynia; hyperalgesia; autonomic dysfunction	-	Low cost and affordability; fast induction of diabetes	High mortality rate; narrow diabetic dose; lack of late neuropathy changes; DN insufficient data
**Diet-induced** (prediabetes/metabolic syndrome)	**High-energy diet** (high-fat, high-sugar, high-fat-high-sugar diets)					
	Rats [17,29,30] (fat 25–60% and carbohydrates 10–80% of food content)	Decreased sensory conduction velocity	Allodynia; hypoalgesia	Decreased intraepidermal nerve fiber density and corneal nerve fiber length	Suitable to study insulin resistant and prediabetes related neuropathy	Extended time to DN development compared to drug-induced DN; lack of frank hyperglycemia
	Mice [26,30,31,32,33] (fat 25–60% and carbohydrates 10–80% of food content)	Decreased sensory and motor conduction velocity	Allodynia; early hyperalgesia, late hypoalgesia	Decreased intraepidermal nerve fiber density	Suitable to study insulin resistant and prediabetes related neuropathy	Extended time to DN development compared to drug-induced DN; lack of frank hyperglycemia
**Drug and diet combined** (type 2 diabetes)	**STR (low doses to moderate) + high-energy-diet**					
	Rats [29,30,34,35]	Slow sensory and motor conduction velocity	Hyper- or hypoalgesia; allodynia	Decreased intraepidermal nerve fiber density; reduction in corneal nerve fiber length; axonal swelling and degeneration; lymphocyte infiltration; Schwann cell damage and demyelination	Shorter time to DN development compared to matched mice; less toxic; gradual development of type 2 diabetes and neuropathy changes	Extended time compared to drug-induced DN development
	Mice [30,35]	Slow sensory and motor conduction velocity	Hypoalgesia	Decreased intraepidermal nerve fiber density	Less toxic; gradual development of type 2 diabetes and neuropathy changes	Extended time compared to drug-induced DN development

**Table 2:** Differences between NCX1, NCX2 and NCX3.

Feature	NCX1	NCX2	NCX3
Tissue Distribution	Heart, skeletal muscle, brain	Brain, retina, kidney	Skeletal muscle, brain
Main Function	Ca^2+^ extrusion in muscle, heart	Ca^2+^ regulation in neurons, glial cells	Ca^2+^ regulation in skeletal muscle
Pathophysiological Role	Heart failure, arrhythmias	Neurodegenerative diseases, synaptic dysfunction	Muscular disorders, neurodegeneration

## Data Availability

The data are available from the authors upon request.

## Abbreviations

The following abbreviations are used in this manuscript:

DN	diabetic neuropathy
PDN	painful diabetic neuropathy
NCX	Na^+^/Ca^2+^ exchanger
NCXrev	NCX reverse mode
STZ	streptozotocin
GLUT	glucose transporter
Ca^2+^	calcium
NMDA	N-methyl-D-aspartate
AMPA	alpha-amino-3-hydroxy-5-methylisoxazole-4-propionicacid
